# A novel proteolytic event controls Hedgehog intracellular sorting and distribution to receptive fields

**DOI:** 10.1242/bio.024083

**Published:** 2017-03-15

**Authors:** Joseph R. Daniele, Tehyen Chu, Sam Kunes

**Affiliations:** Department of Molecular & Cellular Biology, Harvard University, 16 Divinity Avenue, Cambridge, MA 02138, USA

**Keywords:** Hedgehog, Axon transport, Visual system, Apical/basal polarity

## Abstract

The patterning activity of a morphogen depends on secretion and dispersal mechanisms that shape its distribution to the cells of a receptive field. In the case of the protein Hedgehog (Hh), these mechanisms of secretion and transmission remain unclear. In the developing *Drosophila* visual system, Hh is partitioned for release at opposite poles of photoreceptor neurons. Release into the retina regulates the progression of eye development; axon transport and release at axon termini trigger the development of postsynaptic neurons in the brain. Here we show that this binary targeting decision is controlled by a C-terminal proteolysis. Hh with an intact C-terminus undergoes axonal transport, whereas a C-terminal proteolysis enables Hh to remain in the retina, creating a balance between eye and brain development. Thus, we define a novel mechanism for the apical/basal targeting of this developmentally important protein and posit that similar post-translational regulation could underlie the polarity of related ligands.

## Abstract (precis)

**Summary:** Hedgehog is a morphogen and oncogene, but what controls its distribution to release sites? We describe a novel, conserved, proteolytic event in Hedgehog processing that controls delivery to target cells.

## INTRODUCTION

The Hedgehog (Hh) family encodes secreted morphogens with roles in patterning, stem cell maintenance and neoplastic disease (Jiang and Hui, 2008; Scales and de Sauvage, 2009; Varjosalo and Taipale, 2008). A unifying and unresolved question concerning these activities is how they are shaped by the secretion and transport mechanisms that deliver Hh to receptive cells. A number of recent studies have documented the important role of secretory targeting in Hh activity (Briscoe and Thérond, 2013). Hh is released apically or basally in large multimeric or small monomeric forms, which are believed to act as long- and short-range signals, respectively (Ayers et al., 2010; Gallet et al., 2003, 2006; Panáková et al., 2005). The interplay between apical and basal release mechanisms can be complex and interdependent (Callejo et al., 2011). Moreover, it has become clear that patterning previously thought to rely on diffusion in extracellular space might instead involve actin-based cellular extensions (e.g. cytonemes) that transport Hh over many cell diameters prior to release (Rojas-Ríos et al., 2012; and reviewed in Kornberg, 2011).

**Fig. 1.**
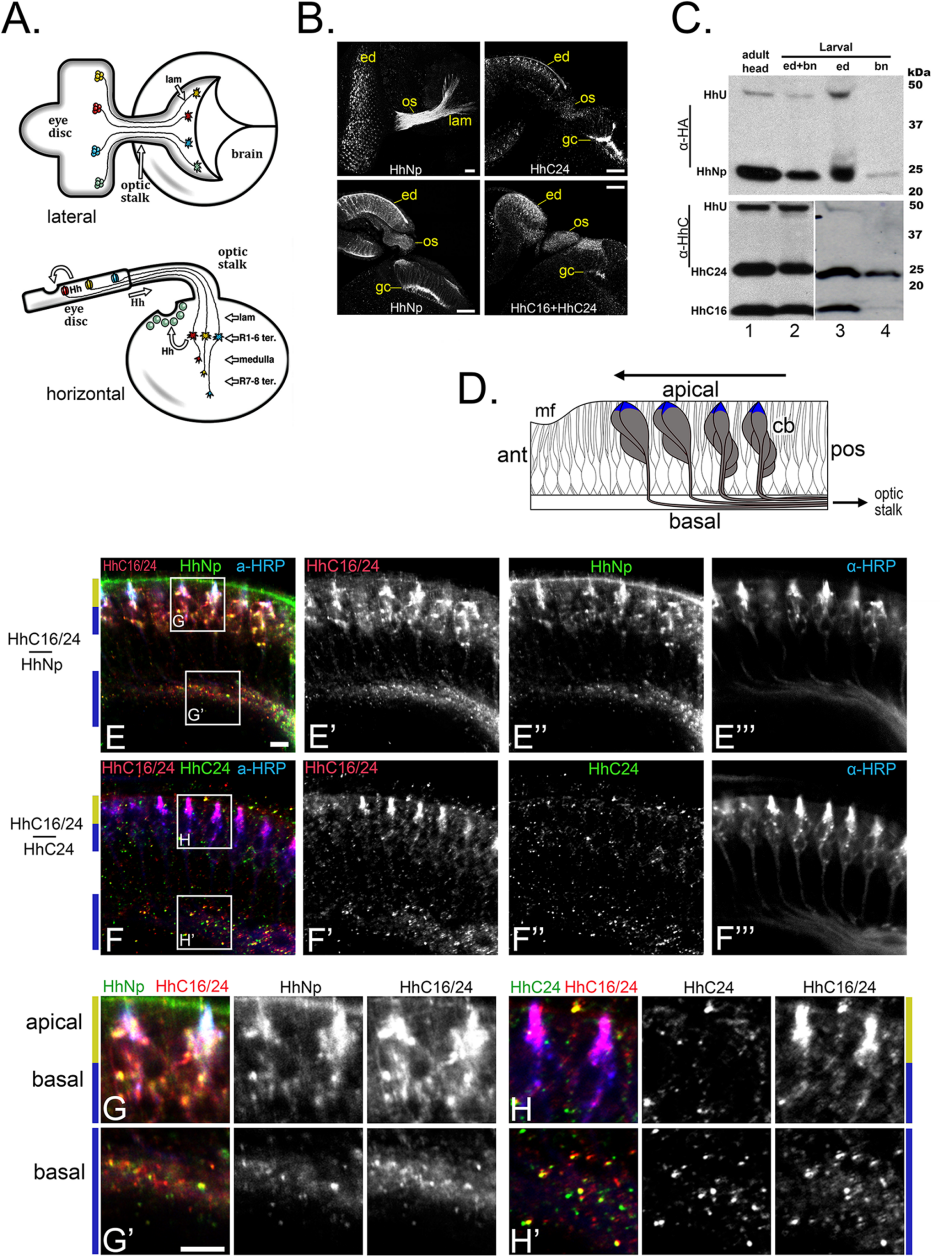
**A long form of the Hedgehog C-terminal domain, HhC, is selectively targeted to photoreceptor axon termini.** (A) The visual system shown from the lateral (top) and horizontal (bottom) perspective. (Top) Photoreceptor neurons differentiate temporally with the posterior-to-anterior progression (right to left) of the morphogenetic furrow across the eye disc. These neurons project their axons (R1–R8) into the brain through the optic stalk, where they spread to dorsal and ventral retinotopic positions (dorsal is up). (Bottom) The R1–R6 axons terminate in the lamina (lam), while R7 and R8 axons terminate in the deeper medulla ganglion. Hh secreted from developing photoreceptor neurons is required for both eye and lamina development. (B) Micrographs showing the distribution of Hh protein (Hh^NHA^ or Hh^CHA3^) expressed with the eye-specific driver *GMR-GAL4*. Scale bar: 20 µm. (Left panels) HhNp visualized with α-HA antibody staining from the lateral (top left) and horizontal (bottom left) perspectives, as described in A. HhNp (derived from Hh^NHA^) is sequestered in puncta in the retinal cell bodies (ed), axons in the optic stalk (os), and growth cones (gc) in the lamina. (Right panels) HhC visualized using either anti-HA antibodies that recognize only full length HhC_24_ (derived from Hh^CHA3^; top right) or anti-HhC antibodies (bottom right), which recognize all HhC polypeptides (HhC_16_ and HhC_24_; see C). HhC_24_ signal is highly concentrated at the growth cones (top right) while the HhC_24_/HhC_16_ staining is evenly distributed from cell body to axon termini (bottom right). (C) Western blot analysis was performed on protein extracts from adult heads (lane 1) or the eye/brain complex of third instar larvae (larval; lanes 2-4) expressing the Hh^NHA^ polypeptide with the *GMR-GAL4* driver. In lanes 3 and 4, the eye/brain complex was dissected to separate the eye disc (ed, lane 3) from the optic stalk and brain (bn, lane 4). Only a small fraction of material is detected as uncleaved precursor product (lane 3, top); nearly all Hh in the brain is self-cleavage product (lane 4, top panel). Uncleaved Hh (HhU), HhC_24_, and HhC_16_ are detected in the eye disc (lane 3, bottom), nearly all HhC in the brain isolate is HhC_24_ (lane 4, bottom). (D) Horizontal schematic of the eye disc showing photoreceptor cell bodies (cb, gray) with their apical tips highlighted (blue). Differentiation proceeds in a posterior (pos) to anterior (ant) wave (direction indicated by arrow), with the onset of ommatidal development on the left at the morphogenetic furrow (mf), and more advanced ommatidia at the posterior (right). (E,F) Horizontal views of the late third instar visual system in animals expressing *UAS-hh^CHA3^* (E) or UAS-*hh^NHA^* (F) under the control of the pan-neural driver *elav-GAL4*. Larval brains were stained with anti-HhC (to visualize all HhC isoforms) or anti-HA antibodies to visualize HhN (E) or HhC_24_ (F). Anti-HRP stains all neuronal membranes and is concentrated at the apical tips of the photoreceptors (see blue color in D). HhC_24_ is concentrated in basal puncta (F and F″) while combined anti-HhC staining reveals strong apical labeling (E′ and F′) from HhC_16_. Puncta labeled by HhNp (E″, anti-HA staining) overlap all HhC (HhC16 and HhC24) stained puncta (see boxed areas in E and F). (G,H) High magnification images of the boxed areas in E (in G,G′) and F (in H,H′). HhNp colabels HhC-positive puncta at the apical tips of photoreceptors (top, G) and axons extending towards the optic stalk (bottom, G′). HhC_24_ (H,H′) is absent from the apical tips (H), unlike combined HhC staining. HhC_24_ is concentrated in axons extending towards the optic stalk (H′). Scale bars: 5 µm. A diagram (Fig. S1) and table (Table S1) of all Hh constructs can be found in the Supplementary materials. All micrographs are representative of three biological replicates.

The central role of secretory mechanisms in Hh activity is illustrated by its segregation between two receptive fields in the developing *Drosophila* compound eye. Hh is synthesized by differentiating photoreceptor neurons and released both apically into the retina, where it propagates a developmental wave of retinal differentiation, and basally, after transport along photoreceptor axons, into the brain, where it induces differentiation of the photoreceptor's postsynaptic target neurons (Fig. 1A and B; Huang and Kunes, 1996; Roignant and Treisman, 2009). Partitioning Hh for release at opposite poles of the photoreceptor neuron is a critical feature of establishing the coordinated development of synaptic partner neurons and their assembly into a precise neural circuit.

How might Hh be partitioned for release at opposite poles of the photoreceptor neuron? Hh is composed of N-terminal and C-terminal domains that dissociate in a self-catalyzed proteolytic cleavage reaction (Lee et al., 1994). The N-terminal product HhNp, modified by cholesterol during self-cleavage, harbors all known Hh signaling activities (Porter et al., 1996). When synthesized in the absence of the C-terminal domain (and hence lacking cholesterol modification), the N-terminal domain is aberrantly targeted and released selectively into the retina (Chu et al., 2006). We previously described a conserved amino acid signal on the C-terminal domain that can override retinal localization, sending both self-cleavage products down the photoreceptor axons for release into the brain (Chu et al., 2006). The question remains, however, how the C-terminal domain, dissociated by self-cleavage, could control secretory targeting, especially of the N-terminal domain, HhNp.

The expected products of Hh self-cleavage include the 24 kDa C-terminal domain, HhC_24_ (Lee et al., 1994), which harbors the axonal targeting motif near its carboxyl terminus (Chu et al., 2006; Lee et al., 1994). We observed that a significant fraction of HhC in photoreceptor neurons is in the form of a 16 kDa polypeptide (HhC_16_; Fig. 1C), an isoform that has been previously observed (Lee et al., 1994; Mastronardi et al., 2003). Here we show that this shortened HhC isoform lacks the axonal targeting motif and that HhC cleavage controls the distribution of Hh between the developing eye and brain. We show that this binary targeting decision involves a pathway choice. Hh with an intact C-terminus enters the axon and is secreted from growth cone tips into the brain. HhNp associated with the shortened isoform, HhC_16_, takes an apical pathway and is responsible for the progression of retinal development. Thus C-terminal proteolysis allows Hh to remain in the retina, creating a balance between eye and brain development.

## RESULTS

### Selective axon transport of HhC_24_, a long-form of the Hh self-cleavage product

The biosynthetic maturation of Hh includes proteolysis and lipid modification coupled to movement through the secretory pathway. Upon translocation into the endoplasmic reticulum (ER), the N-terminal secretion signal sequence is removed to yield the 46 kDa polypeptide Hh-Uncleaved (HhU; Fig. 1C). HhU undergoes an intramolecular self-cleavage reaction that yields the 19 kDa cholesterol-modified N-terminal HhNp and a 24 kDa C-terminal fragment, HhC_24_ that harbors the self-cleavage catalytic domain. Interestingly, an antibody specific to the C-terminal product (Lee et al., 1994) also recognized a shorter fragment of ∼16 kDa (HhC_16_; Fig. 1C, and see Hh processing diagram in Fig. S1). The two polypeptides, HhC_16_ and HhC_24_, were observed with an additional anti-Hh antibody (Tabata and Kornberg, 1994) and after the expression of Hh isoforms tagged by hemaglutanin antigen (HA) insertion carboxyl-terminal to the self-cleavage site (see below). Moreover, both HhC polypeptides were observed in developing visual system extracts, where only native Hh is expressed (Fig. S2A). A ∼16 kDA polypeptide evidently derived from HhC has been noted previously in *Drosophila* embryo extracts (Lee et al., 1994).

To determine which Hh polypeptides transit photoreceptor axons for secretion into the brain, the developing visual system was examined by immunohistochemistry and western blot analysis. For the latter, the developing eye-brain complex, purified from third larval instar animals, was separated into eye and brain fractions by cutting the optic stalk that serves as the portal for photoreceptor axons to enter the brain (Fig. 1A). As previously reported (Chu et al., 2006), HhU was observed only in the retinal fraction (Fig. 1C), indicating that self-cleavage precedes axon transport. The retinal fraction also contained HhNp and both C-terminal species, HhC_24_ and HhC_16_. In contrast, only HhC_24_ and HhNp were concentrated in the brain fraction; HhC_16_ was virtually absent. To localize HhC_16_ and HhC_24_ in intact tissue, immunohistochemistry was used to detect an epitope-tagged Hh isoform [Hh^CHA3^] that was expressed specifically in the retina. In the construct *hh^CHA3^*, an HA-tag inserted at amino acid 267, in HhC, detects HhC_24_ but not HhC_16_ (see diagram in Fig. S1, ‘Construct #13’ in Table S1, and Fig. S2B and C). In these animals, punctate anti-HA labeling was concentrated in distal axons and growth cones in the brain (Fig. 1B, top right panel). HhC_24_-positive puncta were also found in the basal region of photoreceptor cell bodies and axons in the eye imaginal disc (Fig. 1D,F″ and H′), and absent from the apical membrane of cell bodies (Fig. 1F″,H). In contrast, an anti-HhC antibody that detects both HhC_16_ and HhC_24_ (Fig. 1C, bottom panels) (Lee et al., 1994) revealed puncta strongly concentrated in the apical membrane of photoreceptor cell bodies (Fig. 1E′,F′,G and H), in addition to axons and axon termini (Fig. 1B, lower right panel). The signaling domain, HhNp was co-localized with HhC in puncta in both the apical and basal regions, overlapping the HhC_24_-positive puncta in axons and at axon termini, and the presumptive HhC_16_-positive puncta at the apical tips of the photoreceptor cell bodies (Fig. 1E″,G). Hence, the long and short HhC isoforms displayed subcellular localization of opposing polarity, but were nonetheless co-localized with HhNp in both cases.

### C-terminal cleavage follows Hh autoprocessing in the ER

The two HhC isoforms, HhC_16_ and HhC_24_, display differential targeting to axons and transport into the brain (Fig. 1). We thus considered the possibility that these Hh isoforms might enter distinct intracellular trafficking pathways.

Upon translocation into the ER, Hh's N-terminal secretory signal is removed to yield the 46 kDa HhU (Fig. 1C). HhU self-cleavage to yield HhNp and HhC_24_ is thought to occur in the ER (Campbell et al., 2016; Chen et al., 2011). To determine how HhC_24_ and HhC_16_ are processed, we employed Hh-expressing cell lines which enabled us to observe the generation and degradation order of Hh products, the secretion of each fragment, and the organellar compartments in which these events occur. These approaches defined the ER as the site of HhC_16_ generation and demonstrate two distinct secretory outcomes for the HhC fragments

**Fig. 2.**
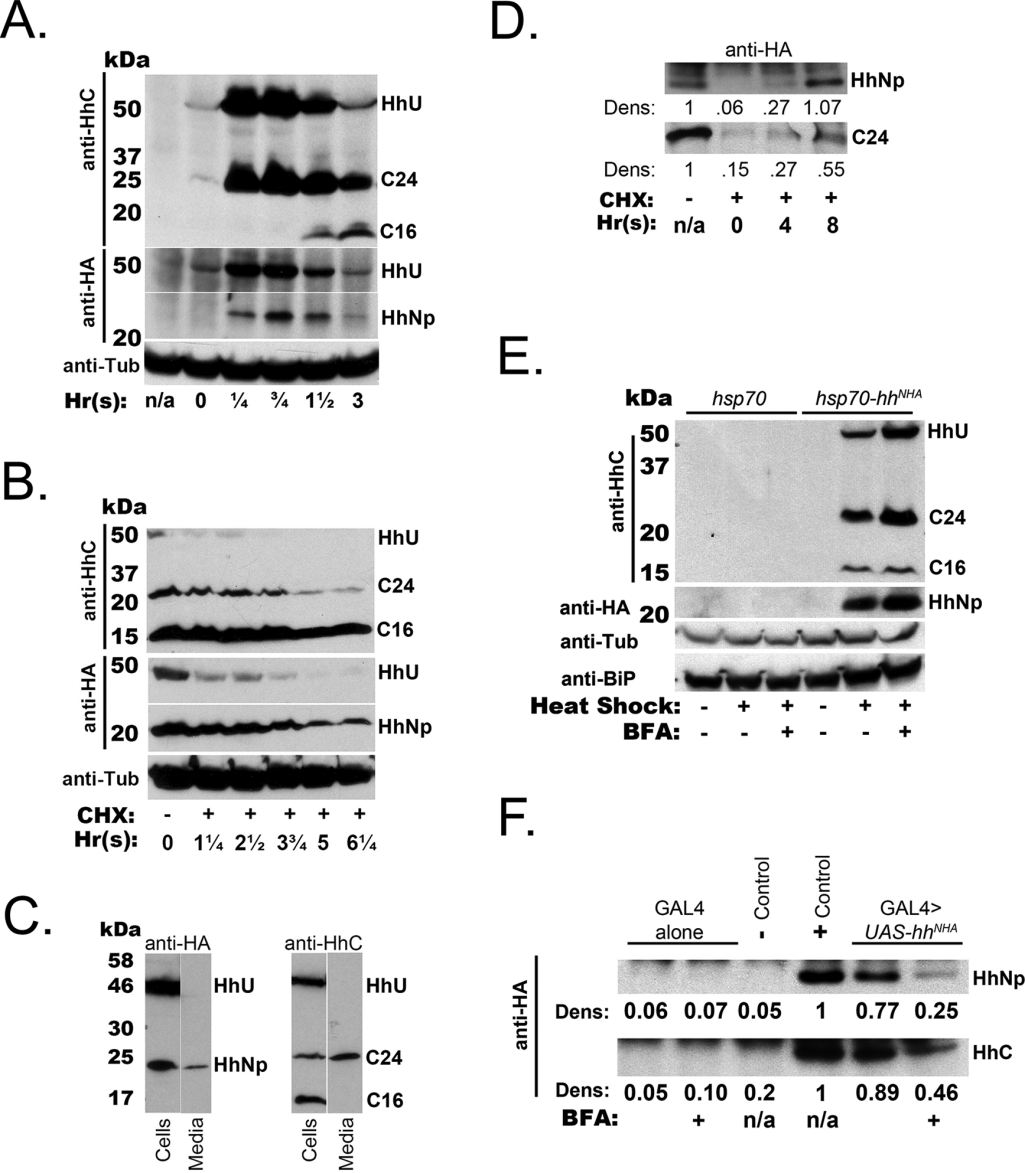
**Differential formation and export of HhC isoforms.** (A) Cells transfected with a heat-shock inducible *hh^NHA^* construct, *hsp70-hh^NHA^*, were subject to a brief heat pulse, after which cell lysates were prepared at the indicated times. ‘n/a’ indicates no heat pulse and ‘Time 0’ lysate was taken at the conclusion of the heat pulse. Western blots were probed with the indicated antibodies. Bands are marked as follows: C24, HhC_24_; C16, HhC_16_. A loading control was visualized with α-Tubulin (anti-Tub) antibody. (B) The translational inhibitor cycloheximide (CHX) was added to cells stably expressing *tub_a1_-GAL4>UAS-hh^NHA^* at time 0 [in hours, ‘Hr(s)’]. Cell lysates were prepared at the indicated time points after CHX addition. Western blot analysis and notation for Hh polypeptides are as in A. The levels of HhNp and HhC_24_ steadily decline while HhC_16_ remains relatively unchanged. (C) Cells expressing *tub_a1_-GAL4>UAS-hh^NHA^* were washed with fresh media and incubated for 3 days. Cell pellets and equivalent amounts of total protein from media were analyzed for HhNp (left panels) and HhC isoforms (right). Note that HhC_16_ is absent from the media fraction. (D) Cells were stably transfected with *tub_a1_-GAL4>UAS-hh^NHA^* (top panel) or *tub_a1_-GAL4>UAS-hh^CHA2^* (bottom panel) and treated with CHX media, as in B. Media was collected after CHX addition at the indicated times, after which the HA-tagged HhNp or HhC was concentrated by immunoprecipitation and visualized by western blot. Quantification by densitometry is shown as the ratio of Hh species in the media relative to media after 3 days incubation with transfected cells (without CHX addition, leftmost lane). HhNp and HhC_24_ appear in the media with similar kinetics to their depletion in CHX-treated cells (B). (E) Brefeldin A (BFA; 20 µM) was added to the culture media of *hsp70-hh^NHA^* transfected cells, after which the cells were treated to a heat pulse. After 3 h, the cells were lysed to prepare extracts for western blot analysis. α-HA staining was used to visualize HhNp. α-Tubulin (anti-Tub) level was measured as a loading control. BiP was examined (anti-BiP) to measure induction of the Unfolded Protein Response by either the heat pulse or BFA treatment. (F) Export of HhNp and HhC_24_ into media was assessed as in D, using immunoprecipitation to concentrate Hh polypeptides from culture media. For BFA addition, BFA was added to *tub_a1_-GAL4>UAS-hh^NHA^* expressing cells for 1 h. Cells were then washed and fresh media with BFA was added. Media was collected after 8 h and examined by immunoprecipitation for HhNp (anti-HA) and HhC (anti-HhC) by western blot. Densitometry is displayed for band intensity relative to the ‘(+) control’ band, for which media was collected after 3 days exposure to *tub_a1_-GAL4>UAS-hh^NHA^* expressing cells. HhNp and HhC_24_ in the media were reduced in the presence of BFA. All blots are representative of three biological replicates.

We first examined Hh processing in a *Drosophila* larval CNS-derived cell culture system that recapitulates proteolysis yielding the three products: HhNp, HhC_24_, and HhC_16_ (Fig. 2A). These products were also observed in an eye-antennal disc-derived cell line and the S2 line (data not shown). A short pulse of *hh^NHA^* expression induced from a heat-shock cassette (*hsp_70_-hh^NHA^*) resulted in the appearance of HhU after a 20-min heat shock. The self-cleavage products HhNp (HA-tagged) and HhC_24_ were coincidently detected in significant amounts at 15 min after heat-shock induction (Fig. 2A). However, another hour passed before HhC_16_ was detected (Fig. 2A). This indication that HhC cleavage follows self-cleavage is consistent with the observation that self-cleavage mutants *hh^C258A^* and *hh*^*441STOP*^ did not produce shortened isoforms consistent with C-terminal cleavage in the absence of self-cleavage (Fig. S3A). Self-cleavage may thus precede and indeed be required for HhC_16_ formation.

The signaling domain, HhNp, is secreted and, when expressed in cultured cells, accumulates in the media (Fig. 2C) (Lee et al., 1994; Maity et al., 2005). Consistent with export, pulse induction of *hh^+^* expression (Fig. 2A) resulted in transient accumulation of intracellular HhNp that peaked at 45 min post-induction. The intracellular level of HhC_24_ displayed similar kinetics (Fig. 2A). The conversion of HhC_24_ to HhC_16_ could account for the reduction in HhC_24_ level at later time points. Surprisingly however, HhC_24_, like HhNp, accumulated in the media of cells transiently expressing *tub _α1_-GAL4>UAS-hh^+^* (Fig. 2C); in contrast, HhC_16_ was not detected in the media. To further resolve the kinetics of Hh processing and secretion, translation in *tub_α1_-GAL4>UAS-hh^+^* transfected cells was blocked with cycloheximide addition to the media (Fig. 2B). By the first time point after cycloheximide addition (1.25 h), HhU was nearly undetectable (>10-fold reduction). The intracellular levels of HhC_24_ and HhNp declined more slowly to ∼50% by 2.5 h after cycloheximide addition. In contrast, the intracellular HhC_16_ level was constant for at least 6 h. HhC_24_ and HhNp coincidently appeared in the media (Fig. 2C), where their concentrations increased at rates inversely corresponding to their diminishing intracellular levels (Fig. 2D). The control nuclear protein Elav was found only in the cell lysate, indicating that HhC_24_ release was not a consequence of cell rupture or death (data not shown). Thus, HhC_24_ and HhNp were released from cells in a temporally coincident and quantitatively similar manner, while HhC_16_ was stably contained within the cells.

To place self-cleavage and HhC cleavage into a subcellular context, we first examined the formation of the proteolytic products in the presence of the toxin Brefeldin A (BFA), which disrupts COPI-mediated ER to Golgi transport and Golgi to ER recycling (Lippincott-Schwartz et al., 1989). Western blot analysis revealed that the level of HhC_16_ was unchanged when BFA was added prior to the induction of *hh* expression from a heat-shock cassette (Fig. 2E). However, the intracellular levels of HhC_24_ and HhNp both increased (HhC_24_, 4.1-fold; HhNp, 1.8-fold). Notably, neither heat-shock nor the addition of BFA increased levels of the ER chaperone BiP, a standard marker for induction of the unfolded protein response (Fig. 2E and data not shown) (Ryoo et al., 2007; and reviewed by Walter and Ron, 2011). In the absence of BFA, transient *hh^+^* expression resulted in contemporaneous accumulation of HhNp and HhC_24_ in the media, while HhC_16_ and HhU remained in the cells (Fig. 2C,F). In the presence of BFA, the export of HhC_24_ and HhNp was greatly diminished (Fig. 2F). Hence, neither self-cleavage nor C-terminal cleavage required COPI-mediated transport to the Golgi. However, cellular export of both HhNp and HhC_24_ were COPI-dependent.

To further clarify which Hh products enter the Golgi apparatus, we engineered a Hh isoform with insertion of an N-linked glycosylation site. The isoform was examined for proteolytic processing and Golgi-specific modification that rendered attached carbohydrate moieties resistant to trimming by endoglycosidase H. Cell lysates obtained from *Drosophila* cell culture expressing a *hh* gene bearing such a site created by a Lys_340_ to Asn substitution were treated with endoglycosidase H (EndoH; Fig. S3B). HhU was entirely EndoH-sensitive (Fig. S3B) which is consistent with its self-cleavage being independent of COPI-mediated ER to Golgi transport (Fig. 2E). In contrast, approximately 75% of HhC_16_ was EndoH-sensitive (Fig. S3B), consistent with its production in the ER. Surprisingly, HhC_24_ was entirely EndoH-sensitive (Fig. S3B) even though Hh is believed to traverse the Golgi. While the absolute EndoH sensitivity of HhC_24_ has been reported previously (Bumcrot et al., 1995), it is possible that the protein's secondary structure might prevent Golgi-specific modification and thus, would lead to these results.

In summary, these experiments indicate that HhC_16_ and HhC_24_ move apicially and basally, respectively, in larval photoreceptors after their formation in the ER.

### Proteolytic cleavage at the Hh C-terminus

**Fig. 3.**
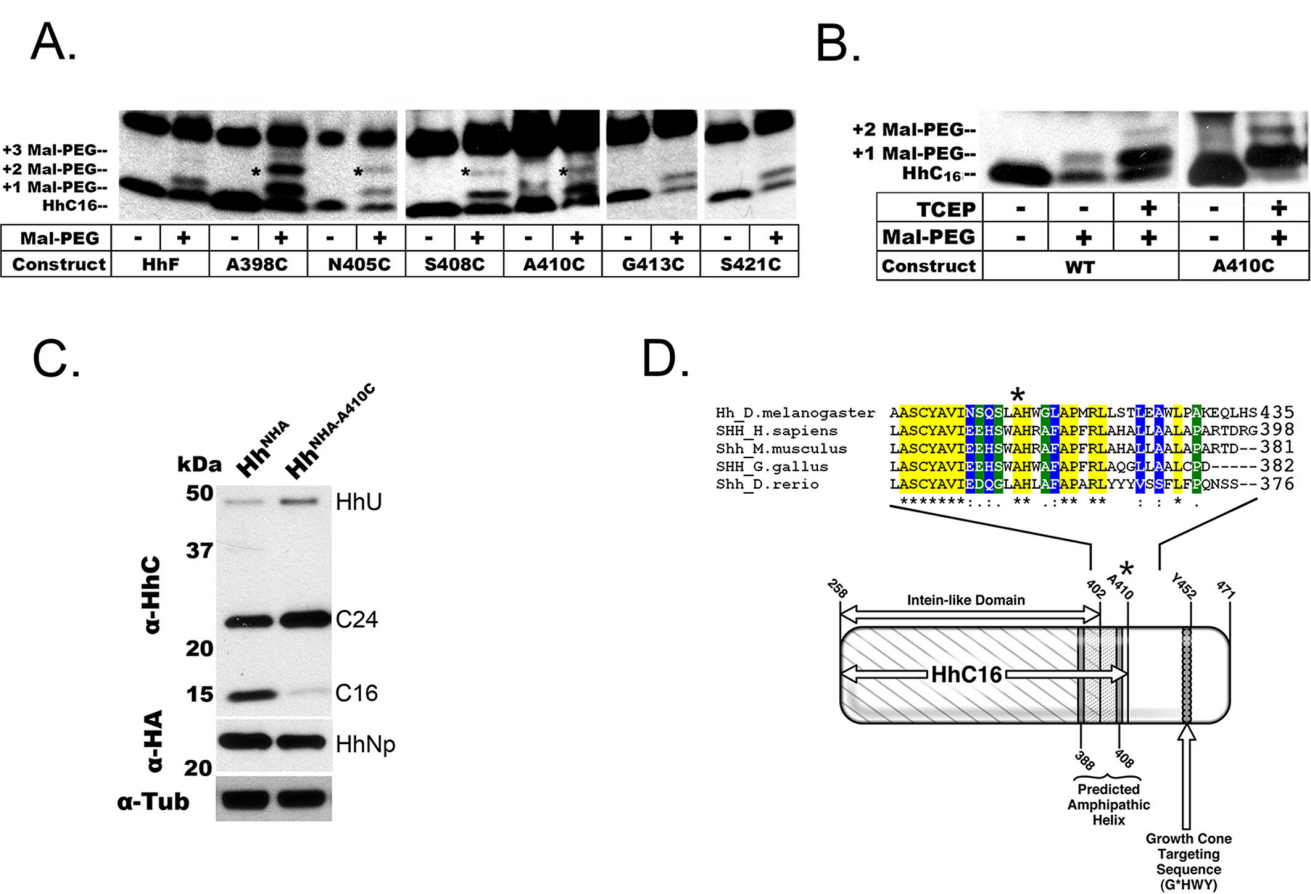
**Localization and mutation of the HhC proteolytic cleavage site.** (A) Cultured cells expressing various *hh* constructs [wildtype (HhF), or with cysteine substitutions at the indicated residues] were lysed and heat-denatured under reducing conditions (e.g. with addition of TCEP; see B). Free cysteines were modified with 1-kDa maleimide-polyethylene glycol (MAL-PEG), as indicated. The western blot (α-HhC staining) reveals the position of HhC_16_ and bands corresponding to +1 MAL-PEG or +2 MAL-PEG modification (asterisk). (B) Maleimide (MAL-PEG) addition and western analysis was performed, as in (A), on lysates from adult *Drosophila* heads expressing the proteins Hh^NHA^ or Hh^A410C^ under control of the eye-specific driver *GMR-GAL4*. Addition of TCEP reduces the disulfide bridge between Cys_258_ and Cys_400_. The considerably stronger MAL-PEG addition after TCEP treatment indicates that most native HhC_16_ bears this disulfide. Substitution of Cys at Ala_410_ creates a novel site for MAL-PEG addition, increasing the intensity of the MAL-PEG addition bands and decreasing the level of native HhC_16_. (C) Western blot analysis of Hh^NHA^ and Hh^A410C^ mutant in adult transgenic animals, with eye-specific expression driven by *GMR-GAL4*. Self-cleavage is evidently normal in the mutant (*hh^A410C^*), as indicated by the relatively normal levels of HhU and HhNp (α-HA, middle). The level of HhC_16_ is strongly reduced, while the alternative isoform, HhC_24_, is increased. (D) Hedgehog family members from several species were aligned using ClustalW. The residue Ala_410_ in *Drosophila melanogaster* (*Dmel*) was conserved in all cases (‘*’ at top). Differing degrees of conservation to either side were classified as fully conserved (yellow, ‘*’), a ‘strong’ association group (blue, ‘:’), or a ‘weak’ association group (green, ‘.’). In cartoon (bottom) labeled domains were identified either previously (e.g. ‘Intein-like Domain’ and ‘Axonal Targeting Motif’) or by hydrophobicity/amphipathic helix prediction (see Fig. S4B and C). The axonal targeting motif was defined by mutation at Tyr_452_ (see Chu et al., 2006). All blots are representative of three biological replicates.

Hh's 9 kDa C-terminal ‘tail’ is thought to be structurally disordered and sensitive to proteolytic attack (Hall et al., 1997). If HhC_24_ were shortened to HhC_16_ by the removal of its tail, it would lack the axonal targeting motif (G*HWY) (see Fig. 3D) (Chu et al., 2006). The loss of this motif would account for the absence of HhC_16_ from photoreceptor axons and the brain (Fig. 1C), consistent with the lack of Hh axon transport in transgenic and genomic mutants deleting a similar region of the Hh C-terminus (Chu et al., 2006). To determine if HhC_16_ is a C-terminally shortened form of HhC_24_, we mapped the cleavage site with maleimide-PEG (mal-PEG) targeted addition (Vitu et al., 2010) and performed size comparison to engineered Hh C-terminal truncations. These approaches defined the span between amino acids 410 and 413 as the site where cleavage yields HhC_16_. The HhC_16_ product would thus lack the axonal targeting motif.

In the mal-PEG method, a 1.0 kDa mal-PEG moiety is added to extracted polypeptide at Cysteine (Cys) residues, and then size-resolved by western blot analysis. The self-cleavage product HhC_24_ has two native Cys residues (Cys_258_ and Cys_400_); if these two residues were present in HhC_16_, mal-PEG addition at either or both would increase the molecular weight of HhC_16_ by 1.0 or 2.0 kDa, respectively. Mal-PEG additions were considerably stronger following the addition of TCEP, which reduces the disulfide bridge between Cys_258_ and Cys_400_, and indicated that most native HhC_16_ contains this bond (Fig. 3B). These two novel bands were indeed observed with the expression of a wild-type *hh^+^* transgene in both cell culture and the adult eye (Fig. 3B; data not shown), indicating that both Cys_258_ and Cys_400_ are contained within HhC_16_. The product with two mal-PEG moieties was however under-represented, likely due to inefficient addition in the basic environment created by adjacent Tyr_401_ and Cys_400_.

For further detailed mapping, a series of constructs was created with single Cys substitutions for amino acids to either side of Cys_400_. These were expressed in a *Drosophila* cell culture system in which HhC_16_ was efficiently produced from a full length Hh transgene (Fig. 3A). Cys substitutions at Ala_398_, Asn_405_, Ser_408_, and Ala_410_ resulted in HhC_16_ species that were modified at the novel Cys residue (Fig. 3A). Moreover, Ala_410_ resulted in HhC_16_ species that were modified at the novel Cys residue when expressed in the retina of transgenic animals (Fig. 3B). In contrast, Cys substitution at Gly_413_ or Ser_421_ did not introduce a novel mal-PEG modifiable site into HhC_16_ (Fig. 3A). To confirm localization of the cleavage between Ala_410_ and Gly_413_, we engineered a series of HhC truncations by inserting a start codon at the self-cleavage site (Cys_258_) and stop codon at various carboxyl-terminal sites expected to produce a polypeptide of 16-17 kDa. The truncated polypeptide produced by a stop codon at Leu_414_ had slightly slower gel mobility than HhC_16_, while other nearby truncations created products with larger differences in mobility (Fig. S4A). These results indicate that HhC_16_ is generated by cleavage between residues Ala_410_ and Gly_413_. Notably, Cys substitution at Ala_410_ strongly reduced HhC_16_ formation in cultured *Drosophila* cells (∼53% reduction; Fig. 3A). When the same mutant Hh protein was expressed in the retina of transgenic animals, self-cleavage to yield HhNp occurred normally (Fig. 3C), but the C-terminal fragment accumulated as HhC_24_, while HhC_16_ was barely detectable (90% reduction; *hh^NHA-A410C^*). This also indicates that self-cleavage does not require cleavage at this second cleavage site in order to produce mature HhNp. Alignment of Hh from diverse species revealed that the amino acid sequence surrounding Ala_410_ is well conserved (Fig. 3D). Interestingly, the HhC cleavage site is adjacent to a hydrophobic amphipathic helix (Fig. 3D; Fig. S4B and C), which suggests possible association of this domain with hydrophobic membranes and substrates and a potential remodeling/refolding of this hydrophobic stretch when C-terminal cleavage occurs (Hall et al., 1997). Further, such regions are common among proteins associated with apolipoprotein particles, the reported vehicle of Hh transport (Smolenaars et al., 2007; Panáková et al., 2005).

### C-terminal cleavage controls Hh spatial localization and targeted signaling activity

**Fig. 4.**
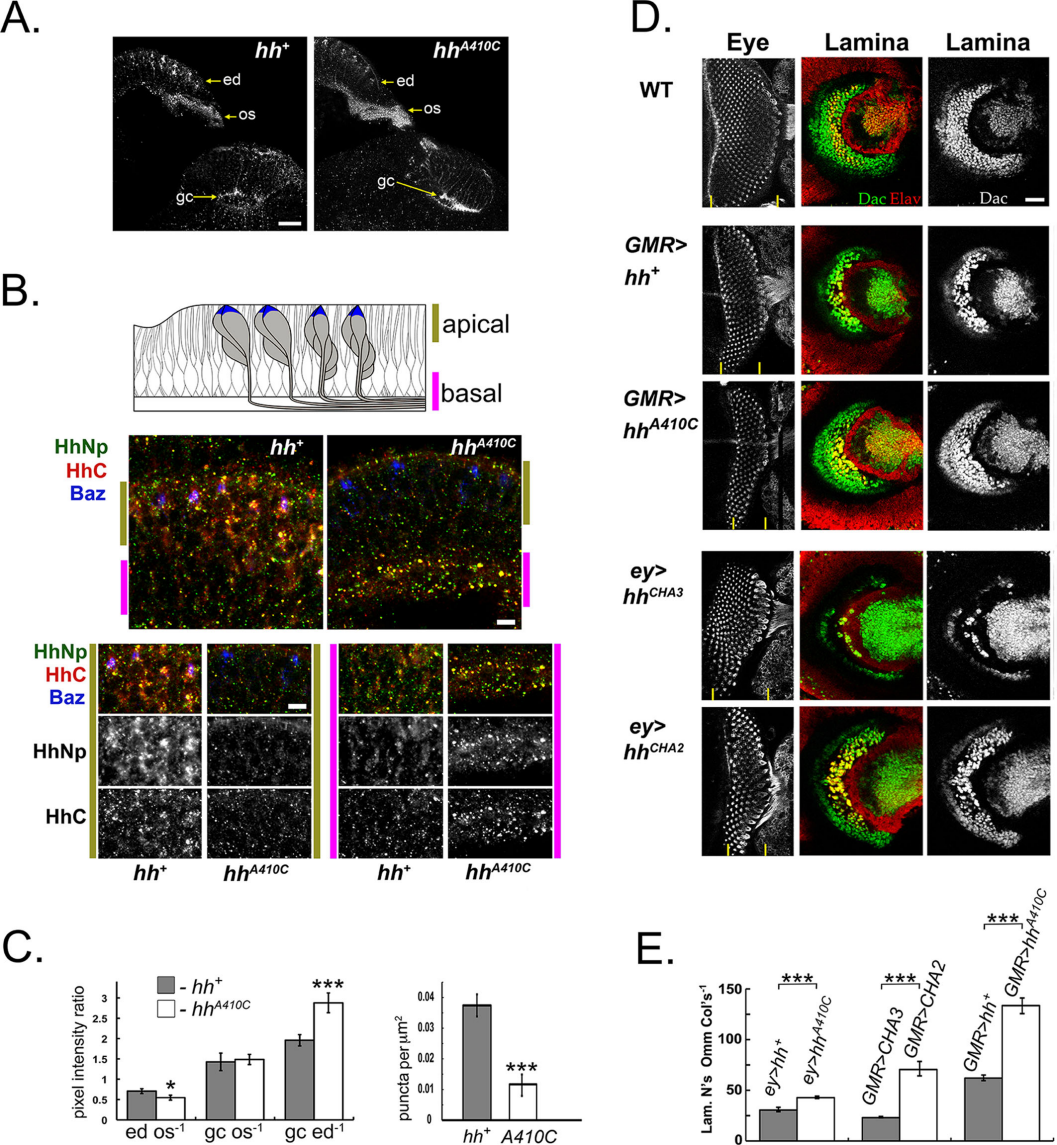
**C-terminal cleavage controls the polarity of Hh localization and the balance of eye and brain development.** (A) Horizontal perspective of the developing eye, brain complex at late third instar stage, comparing wild-type *hh^+^* (left) protein localization to the HhC cleavage mutant, *hh^A410C^* (right). Transgenes were expressed with the pan-neural driver *elav-GAL4*. HhNp localization (α-HA staining) is reduced in the apical retina (ed) and enhanced in the optic stalk (os) and at photoreceptor R1-6 growth cones (gc) in the *hh^A410C^* mutant. Scale bar: 20 µm. (B) Higher magnification view (than in A) comparing apical, basal localization of HhNp and HhC in the wild-type (*hh^+^*) and *hh^A410C^* mutant. The coalesced apical tips of photoreceptor cells in an ommatidium were marked with Bazooka::GFP (Baz, blue color). Note that both HhNp (anti-HA staining, red color) and HhC (green color) are strongly reduced in the apical region of animals expressing Hh^A410C^ (right middle panel). Green and magenta bars indicate the apical and basal regions, respectively, examined in higher magnification views in the bottom panels. The apical region of the Hh^A410C^ mutant has much less HhNp and HhC staining (panels demarcated by green bars), while the basal region composed of photoreceptor axons has greater HhNp and HhC staining, as co-labeled puncta in the Hh^A410C^ mutant (panels demarcated by magenta bars). Scale bar: 20 µm. (C) Quantitative analysis of Hh distribution in the wild type and C-terminal cleavage mutant. (Left) Quantification of HhNp in the retina (ed), optic stalk (os), and growth cone (gc; as described in Chu et al., 2006) based on average pixel intensity measurements (see Materials and Methods). The ratios of pixel intensity measurements were calculated, as indicated. Error bars indicate s.e.m. *P<0.05, ***P<0.001 by two-tailed *t-*test. (Right) The average number of HhNp-positive apical puncta was quantified per unit area after expression of the wild type (*hh^+^*) and mutant (*hh^A410C^*) transgenes. Plots are representative from three biological replicates. Data collected from: *hh^NHA^ n*=13 and *hh^A410^*^C^ *n*=12 specimens. Error bars indicate s.e.m. ***P<0.001 by two-tailed *t-*test. (D) Rescue of eye and lamina development by eye-specific transgene expression in the *hh^1^* genetic background. Approximately 11 ommatidial columns are formed in the visual system-specific regulatory mutant *hh^1^*. Lamina induction, measured by the formation of Dachshund (Dac)-positive lamina precursor cells (α-Dac, green color) and Elav-positive lamina neurons (α-Elav, red color) is completely absent in the mutant (*hh^A410C^*) (not shown; Huang and Kunes, 1996). Representative late third instar specimens are shown, with corresponding eye and brain micrographs (lateral perspective). Ommatidial columns (left panels) were revealed by α-HRP staining (grayscale). Regions of ommatidial development are marked by vertical yellow bars at the bottom of each image (left panels). With the strong driver *GMR-GAL4*, GAL4 activity was attenuated with the temperature-sensitive *tub_α1_-GAL80^ts^* inhibitor (McGuire et al., 2003) employed at a semi-permissive temperature (25°C, as shown). Under these conditions, eye and lamina development with the wild-type transgene (*GMR>hh^+^*) is reduced from *hh^+^* background (top panels). Rescue with the *hh^A410C^* transgene (*GMR>hh^A410C^*) yields fewer ommatidial columns and more lamina precursor cells and lamina neurons. With the weak eye-specific driver *eyeless-GAL4*, a transgene with normal HhC cleavage yields rescue with normal ommatidial development and reduced lamina development (*ey>hh^CHA3^*). With a mutant transgene that lacks HhC cleavage, lamina development is rescued, while eye development is reduced (*ey>hh^CHA2^*). Scale bar: 20 µm. (E) Quantitative analysis of ommatidial development and lamina induction in specimens from experiments shown in D. The average ratio of lamina neurons (Lam. N's) to ommatidial columns (Omm Cols) was determined in 3D reconstructions of confocal micrographs (see Materials and Methods). Significance scores above bars are shown relative to each hh construct with ‘normal processing’. *P<0.05, ***P<0.001 by two-tailed *t-*test. Plot is representative from three biological replicates. Data collected from: *ey>hh*^+^ *n*=8; *ey>hh*^A410C^ *n*=12; *GMR>hh^CHA3^ n*=7; *GMR>hh^CHA2^ n*=9; *GMR>hh^+^ n*=7; and *GMR>hh^A410C^ n*=7 specimens. All micrographs are representative of three biological replicates.

If proteolytic loss of the axonal targeting motif is a determinant of Hh localization in photoreceptor neurons, we would expect a C-terminal cleavage site mutation to shift Hh localization from the developing eye to the brain. Moreover, with visual system development under the control of such a mutant, the induction of lamina cells might increase at the expense of photoreceptor cells. To test these predictions, we quantified immunofluorescence from developing photoreceptor cells, comparing the wild-type localization of HhNp and HhC to two Hh mutants that lack C-terminal cleavage. In the wild type (see also Fig. 1B), HhC isoforms and HhNp are present in both the retina and photoreceptor axons (Fig. 4A,C; Fig. S5A). In contrast, HhNp and HhC derived from Hh^A410C^ were shifted to axon termini (Fig. 4A,C; Fig. S5A). Both HhNp and HhC co-labeled puncta were absent from the apical membranes of photoreceptor cell bodies (Fig. 4B, bottom left panels), where they are normally found in the wild type (Fig. 4B and C, right panel, ∼threefold decrease) in clusters surrounding the apical cell marker Bazooka (Djiane et al., 2005). Conversely, the number of HhNp and HhC co-labeled puncta in photoreceptor axons was markedly increased in the Hh^A410C^ mutant (Fig. 4B, bottom right panels). An HA-tag insertion at Ala_358_ (*hh^CHA2^*) also resulted in a C-terminal cleavage mutant phenotype; it displayed normal self-cleavage without forming HhC_16_ (Fig. S5B). As with *hh^A410C^*, the distribution of HhNp and HhC derived from Hh^CHA2^ was shifted to axon termini (data not shown).

We have shown that eye and lamina development are controlled by the release of Hh from opposite ends of the photoreceptor neuron (Chu et al., 2006). A mutation in the C-terminal axonal targeting motif resulted in HhNp retention in the retina and a deficit in lamina development (Chu et al., 2006). The genomic mutation, *hh^2^*, deleted the axonal targeting motif and displayed a similar lamina phenotype. We reasoned that, if more HhNp is released by each photoreceptor axon that arrives in the brain, HhC cleavage mutants might favor lamina development at the expense of eye development. To address this question, the numbers of lamina precursor cells and ommatidia were quantified when HhC-cleavage mutant transgenes were used to rescue visual system development in a visual system-specific *hh^1^* genetic background. The mutant transgenes *UAS-hh^A410C^* and *UAS-hh^CHA2^* were expressed specifically and at a low level in the developing eye with the *eyeless^116^-GAL4* (*ey-Gal4*) driver or the strong retina-specific driver *GMR-GAL4* in the presence of the regulatory subunit encoded by *tub_α1_-GAL80^ts^* (McGuire et al., 2003) to suppress GAL4 activity. Thus, transgenic *hh* was supplied in limiting amounts [compare *GMR>UAS-hh^+^* or *ey>hh^CHA3^* to the wild type (WT) in Fig. 4D]. We examined the effect on eye and lamina development in late third instar larval animals, before apoptosis eliminated lamina precursor cells that failed to interact with an ommatidial axon fascicle (Huang et al., 1998). In this context, we observed that shifting the polarity of Hh secretion altered the ratio of lamina to retinal development (Fig. 4D,E).

Hh induces the formation of lamina precursor cells, which express the marker Dachshund (Huang and Kunes, 1996; Mardon et al., 1994). We quantified photoreceptor neurons via their expression of the neuronal markers Elav and HRP. The number of lamina precursor cells was quantified in complete Z-stack reconstructions of the brains of late third instar larvae (Fig. 4D,E; data not shown). For each specimen, the corresponding retina was examined to quantify the anterior progression of eye development (Fig. 4D, left panels). Notably, with reduced eye and lamina development in *GMR-GAL4*, *tub_α1_-GAL80^ts^* animals, the ratio of lamina precursor cells to ommatidial columns was increased in Hh^A410C^, relative to wild type Hh (Fig. 4E). Similarly, when either Hh^A410C^ or Hh^CHA2^ was expressed with the weak driver *ey-GAL4*, there were more lamina precursor cells and fewer ommatidia than in the *hh^+^* control (Fig. 4D,E). Thus, converse to deletion or mutation that removes the axonal targeting motif, the loss of HhC cleavage favors lamina development at the expense of retinal development.

## DISCUSSION

The activity of a morphogen depends on the mechanisms of secretion and dispersal that shape its access to cells of a receptive field. This is the case for Hh, whose secretion and transmission is complex and remains unresolved. One view of Hh transmission posits its diffusion in extracellular space as monomeric protein, multimeric complex or in lipoprotein particles. Another view rests on long cellular extensions, filopodia or cytonemes, over which Hh may be carried for many cell diameters. These modes of transmission are not mutually exclusive and indeed may coexist and cooperate to create the spatial shape of the Hh signaling gradient. Resolving the secretory pathways that emit Hh from its cells of origin is key to understanding these modes of transport.

There is ample evidence that one of the determinants of Hh dispersal is polarized secretion (reviewed by Kornberg, 2011; Therond, 2012). A number of models have based the differential range of Hh on selective export from either the apical or basal poles of the cell. For example, work in the developing *Drosophila* wing indicates that apically secreted Hh is reabsorbed and redirected to basal cytonemes, which then transmit Hh in a long-range signaling gradient (Callejo et al., 2011). We have defined a system in which polarized secretion accounts for coordinated developmental programs in the *Drosophila* eye and brain. Apical Hh secretion propagates the temporal wave of ommatidial development in the eye, while basal targeting to photoreceptor axons induces the differentiation of post-synaptic lamina neurons in the brain (see Fig. 1A) (Huang and Kunes, 1996; reviewed by Roignant and Treisman, 2009). We previously defined a small region of the Hh C-terminus that is necessary and sufficient for basal secretion (Chu et al., 2006). Hh lacking this axonal targeting motif is mostly secreted apically, possibly due to an apical targeting signal(s) near the N-terminus (T.C. and S.K., unpublished observations). Here we show that the distribution of Hh between the eye and the brain is controlled by proteolytic cleavage at a site in the Hh C-terminal domain.

The proteolytically shortened HhC_16_, which lacks the axonal targeting motif (Figs 1G,H and 4B) was preferentially localized at the apical tips of photoreceptor neurons in puncta containing HhNp, the developmental signaling domain (Fig. 1C,F). This is consistent with the prior observation that Hh remains in the retina in C-terminal deletion and point mutants that lack the axonal targeting motif (Chu et al., 2006). In contrast, HhC_24_, was found in basally localized particles with HhNp localized in photoreceptor axons and growth cones (Fig. 1B,C,E and G′). HhC_24_ may be released from growth cones, as it is from cultured cells (Fig. 2), though it has no known signaling activity (Roelink et al., 1994, 1995). The shortened isoform HhC_16_ appears to be retained in the cell, at least in culture (Fig. 2), despite entering the Golgi (Fig. S3B). This binary decision evidently controls the distribution of Hh between the developing eye and brain, as the distribution of HhNp was shifted to the brain when C-terminal cleavage was blocked by mutation (Figs 3 and 4). Under conditions of limited Hh synthesis, the shift in the polarity of secretion was matched by enhanced induction of lamina precursor cells in the brain and reduced ommatidial development (Fig. 4). It is possible, then, that in normal development the control of C-terminal cleavage balances Hh's activities between the retina and brain. In this regard, we have identified a regulator of Hh C-terminal cleavage that controls *Drosophila* eye development in a *hh*-dependent manner (J.R.D. and S.K., unpublished data).

**Fig. 5.**
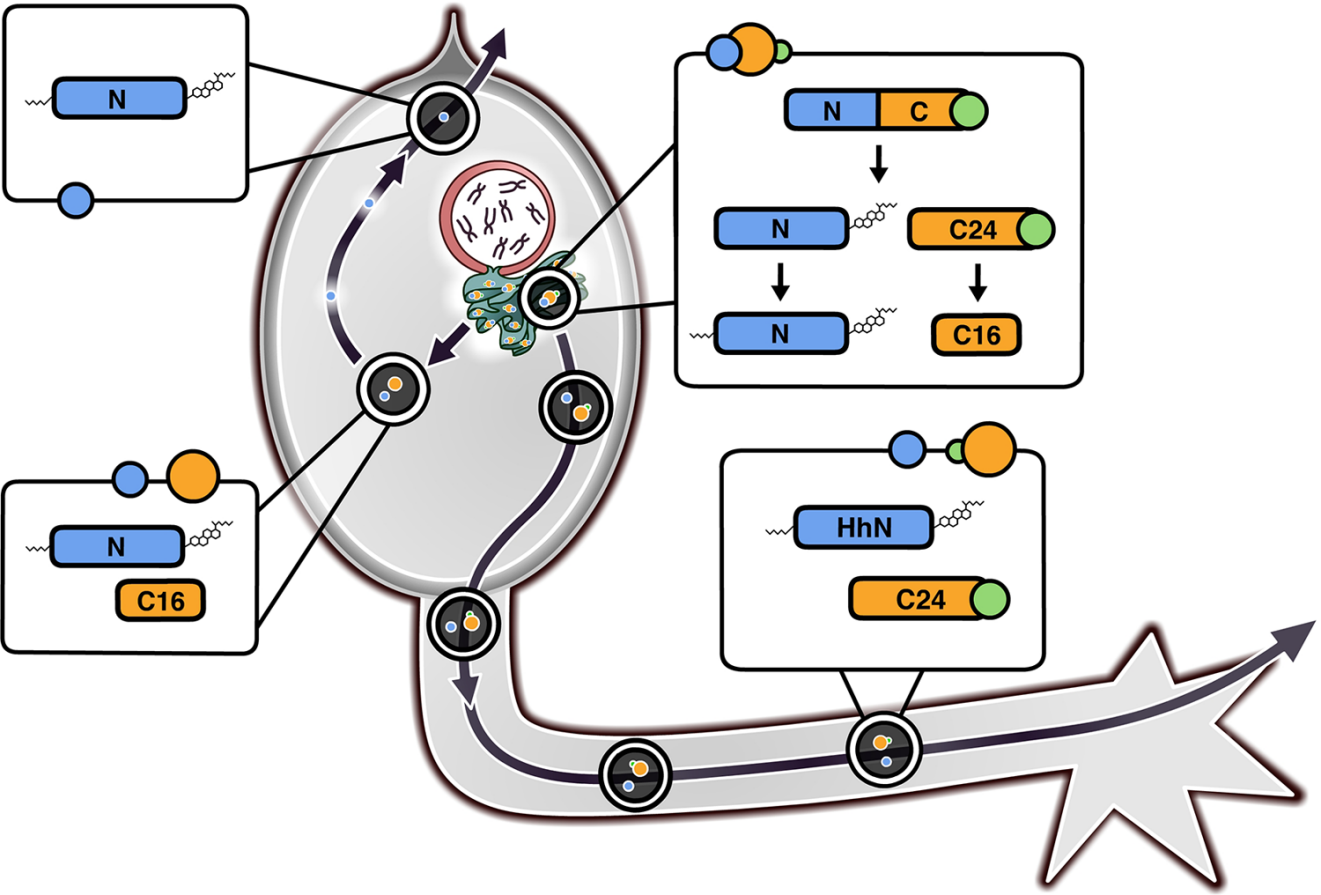
**Hh cleavage products depend on a C-terminal cleavage to determine Hh axonal transport.** Sorting of HhC_24_ and HhC_16_ presumably occurs in the ER where both autocleavage and C-terminal cleavage take place and Hh particles are assembled. ‘HhNp’ is labeled in blue. ‘HhC24’ is labeled in orange and contains the ‘growth cone targeting sequence’ (green circle). HhC16 is also orange but does not possess the targeting sequence. Particles containing HhNp and HhC_24_ bud from the ER and travel down the axon. Particles containing HhNp and HhC16 (which lacks the ‘growth cone targeting sequence’), however, will remain in the photoreceptor cell body and HhNp will be released apically.

Of note, the HhC_24_ cleavage site is adjacent to a hydrophobic amphipathic helix (Fig. 3D; Fig. S4B and C); such regions are common among proteins associated with apolipoprotein particles (Smolenaars et al., 2007). The ER is a likely source of HhC containing particles since it appears to be the locale where Hh cleavage products are formed (Fig. 2). While it has been shown previously that COPI is not necessary for Hh self-cleavage, far less is known about COPI and COPII dependence on Hh secretion (Fig. 2E,F) (Aikin et al., 2012; Chen et al., 2011). In a genome-wide screen for Hh secretion, COPI inhibition appeared to block most Hh export while COPII knock-down had only a modest effect (Aikin et al., 2012). Thus, HhNp and HhC_24_ may be captured in the same particle in this ER-localized process, which leads to their basal targeting and axon transport (Fig. 5). This association between Hh termini is likely mediated in part by the lipophilic moieties on HhNp and the amphipathic tail on HhC24. Cholesterol modification of the mature HhNp ligand, for instance, enables its interaction with lipid raft proteins such as Caveolin (Reggie1) and the putative proton transporter Dispatched (Aikin et al., 2012; Burke et al., 1999; Callejo et al., 2011; Katanaev et al., 2008). In contrast, HhC_16_, possibly associated with HhNp, lacks the axonal targeting motif, and may have its amphipathic helix disrupted by proximity to its novel C-terminus, which results in its apical targeting (Fig. 5). This model is plausible as the stabilization and ‘solubilization’ of the hydrophobic *Dmel* HhC_24_ upon C-terminal proteolysis (of the last ∼9 kD) has been reported previously (Hall et al., 1997). Such a change in the structure of this C-terminal tail could allow HhNp to instead associate with other binding partners and thus, would influence its apical/basal targeting.

Finally, the HhC cleavage site between residues Ala_410_ and Gly_413_, is conserved in diverse Hedgehog family members (Fig. 3D). While it is not clear whether C-terminal cleavage is common, it has been reported for human SHH (Mastronardi et al., 2003). Furthermore, a mutation at this site yields a moderate form of holoprosencephaly (Roessler et al., 2009). It is generally not understood how HhC region mutations yield Hh loss-of-function phenotypes; clearly, defects in secretory targeting are one possibility. Thus we have defined a novel mechanism for the apical/basal targeting of a developmentally important ligand and due to its conservation in humans, it is possible that this same process might underlie the targeting of other post-translationally modified ligands.

## MATERIALS AND METHODS

### Strains and reagents

The *UAS-hh^NHA^* (Burke et al., 1999), *UAS-hh^CHA2^* and *UAS-hh^CHA3^* (Chu et al., 2006) transgenic animals were described previously. The following stocks were obtained from the Bloomington *Drosophila* Stock Center (Bloomington, IN, USA): y,w, GMR-GAL4/CyO; y,w, eyeless-GAL4 (p116#5); y,w, elav-GAL4 (X); w; elav-GAL4 (III); w[*]; tub-GAL80^ts^; w[*], UAS-baz::GFP.

The *GMR-GAL4* and *eyeless-GAL4* drivers were introduced into a *hh^1^* background to either create recombinant chromosomes (e.g. *GMR-GAL4*>*UAS-hh^NHA^*) or to perform genetic eye rescues. Transgenic *Drosophila* (*UAS-hh^NHA-A410C^*) was made by Best Gene (Chino Hills, CA, USA).

### Molecular biology

To construct HhC truncations, primers were made to flank the C258 codon (first codon of HhC) and the last codon of each truncation (e.g. S408, G413). The forward primer substituted an ‘ATG’ start codon for C258 and the reverse primer placed a ‘TAG’ stop codon after the truncation site. Truncations were cloned into *pUAST* with EcoRI and BglII.

For heat-shock inducible *hh^+^*, primers were designed to the HA tagged *hh* (pDA519) to enable its cloning into *pCasSper-hs* (U59056, DGRC, Bloomington, IN, USA) using EcoRI and XbaI (Burke et al., 1999). For the cysteine and the glycosylation site substitutions, *hh^NHA^* was cloned into *pAc5.1B-lambdaN* (22420, AddGene, Cambridge, MA, USA). This *pAc5.1B_hh^NHA^* construct was then mutated using the QuikChange Site-Directed Mutagenesis Kit (Agilent, Santa Clara, CA, USA). Cysteine Substitutions: *pAc5.1B_hh^NHA-A398C^*, ‘GCC’ for A398 was changed to ‘TGC’, a codon for cysteine; *pAc5.1B_hh^NHA-N405C^*, ‘ACC’ for N405 was changed to ‘TGC’; *pAc5.1B_hh^NHA-S408C^*, ‘TCG’ for S408 was changed to ‘TGT’; *pAc5.1B_hh^NHA-A410C^*, ‘GCC’ for A410 was changed to ‘TGT’; *pAc5.1B_hh^NHA-G413C^*, ‘GGA’ for G413 was changed to ‘TGT’; and *pAc5.1B_hh^NHA-S421C^*, ‘TCC’ for S421 was changed to ‘TGC’. To make *pUAST_hh^NHA-A410C^*, *hh^NHA-A410C^* was cut with KpnI and XbaI from *pAc5.1B_hh^NHA-A410C^* and ligated into *pUAST*. For *pAc5.1B_hh^NHA-K340N^*, ‘AAG’ for K340 was changed to ‘AAT’ to create an ‘Nx(S/T)’ glycosylation site.

### Immunohistochemistry

Antibody staining was performed as previously described (Huang and Kunes, 1996). Antibody dilutions: mouse α-Dac (mAbDac2-3, DSHB, Iowa City, IA, USA) 1:10; rat α-Elav (7E8A10, DSHB) 1:25; mouse α-HA (12CA5, Abcam, Cambridge, MA, USA) 1:200; rabbit α-HA, pre-absorbed (Santa Cruz, Dallas, TX, USA) 1:400; rabbit α –HhC, preabsorbed (Lee et al., 1994), 1:400; mouse α-Chaoptin (24B10, DSHB), 1:200; rabbit α-GFP (A11122, Thermo Fisher, Waltham, MA USA) 1:400; mouse α-GFP (A11120, Thermo Fisher) 1:200; Cy3-goat α-mouse (Jackson, Bar Harbor, ME, USA), 1:200; Cy3-goat α-rabbit (Jackson), 1:500; Cy5-goat α-mouse (Jackson), 1:200; Cy5-donkey α-rabbit (Jackson), 1:500.

### Western blot analysis

Western blot samples were prepared and performed as in Chu et al. (2006). Protein concentration was quantified (RC DC Protein Assay, BioRad, Hercules, CA, USA) and equivalent protein was loaded into each well. For samples prepared from *Drosophila* tissues equivalent tissue amounts were loaded. Primary antibodies: rabbit α-HA (Santa Cruz) 1:1000; rabbit α-HhC 1:1000 (Lee et al., 1994); mouse α-tubulin-alpha (AA4.3, DSHB) 1:4000; rabbit α-GFP (A11122, Thermo Fisher), 1:4000; guinea pig α-BiP (provided by Hyung Ryoo, New York University, New York, NY, USA), 1:1000; rabbit α-Hh (provided by Tetsuya Tabata, University of Tokyo, Tokyo, Japan), 1:2000. Secondary antibodies: HRP-conjugated α-rabbit (GE Biosciences, Pittsburgh, PA, USA), HRP-α-mouse (GE Biosciences), HRP-α-rat antibody (Jackson), and HRP-α-guinea pig (Jackson) at a 1:5000 or 1:30,000 dilution.

### Cell culture experiments

ML-DmBG3-c2 cells (DGRC) were cultured in a humidified, 23°C incubator, as previously described (Ui et al., 1994). Qiagen Effectene (Qiagen, Hilden, DE) was used for transfections; 1 mg of DNA per plasmid. Time course: cells transfected 60 h previously with *pCasSper-hs-hh^NHA^* were heat-shocked for 20 min at 37°C (control plate at 23°C) and then placed at 23°C. Cells were lysed at specified times following heat-shock and prepared for western blotting. ER to Golgi block: cells transfected 60 h previously with either *pCasSper-hs* (empty vector) or *pCasSper-hs-hh^NHA^* had Brefeldin A (BFA, Sigma Aldrich, St. Louis, MO, USA) added 1 h before heat-shock (final, 20 µM). One ‘no BFA’ control plate stayed at 23°C. Two ‘heat-shock’ plates, with or without BFA, spent 20 min at 37°C. Plates were then placed for 3 h at 23°C. Then, cells were scraped off plates and prepared for western blots. Immunoprecipitation (IP): cells were transfected 60 h previously with *tub_α1_-GAL4* and *UAS-hh^NHA^* or *UAS-hh^CHA2^* had fresh media added. Cycloheximide (CHX, Sigma Aldrich) was added to plates (final, 50 mg ml^−1^). Media aliquots were collected at specified time points. A modified Dynabeads Protein A (Invitrogen, Carlsbad, CA, USA) IP protocol was used to pull HhN::HA or HhC::HA from media. Briefly, 0.9 ml of cell culture media (spun 1000×g, 5 min) was mixed with an equal volume of chilled, Non-Denaturing Lysis Buffer [NDLB, pH 8; 20 mM Tris HCl pH 8, 137 mM NaCl, 1% NP-40, 2 mM EDTA (pH 8), Protease Inhibitor]. Rabbit α-HA (Y-11 sc-805, Santa Cruz) was then added (final, 0.6 ng ml^−1^) and tubes were nutated overnight at 4°C. In the morning, 75 µl of Protein A beads, washed 3× with chilled NDLB, were added to the media/NDLB/antibody mixture and nutated for 1 h at 4°C. Beads were precipitated with a DYNA I MPC-S magnet, washed 2× with chilled NDLB, and then on the third wash 75 µl Laemmli Buffer (BioRad) (+βME+Protease Inhibitor Cocktail) was added and samples were prepared for western blotting. For the BFA immunoprecipitation experiment, control media was taken from resting transfected cells, after which one set of cells was incubated with BFA (final, 20 µM) for 1 h. After preincubation, cells were washed 2× with fresh media, BFA was reapplied, and cells placed for 8 h at 23°C. Media was then collected from BFA-treated and untreated cells. Following steps were identical to the IP above.

### Maleimide addition

Adapted from Vitu et al. (2010), 10 adult *Drosophila* heads or one plate of transfected cells (expressing *pAc5.1B -hh^NHA^* with or without cysteine substitutions) were homogenized with Laemmli Buffer (-βME, +Protease Inhibitor), placed at 95°C for 5 min, spun down (1000× ***g***, 3 min), and had supernatant transferred to new tubes. TCEP (Sigma Aldrich) was added to each sample (final, 5 mM) for 10 min at 23°C. Sample pH was equilibrated to ∼7 using filtered 1 M KOH and EZ-Link Maleimide-PEG11-Biotin (Thermo Fisher) (or DMSO alone) was added (final, 1 mM). Reactions proceeded for 2 min before being quenched with βME (final, 5%) and allowed to sit for 5 min at 23°C. Samples were stored at –80°C.

### Glycosidase treatment

Endoglycosidase H (Roche, Basel, SUI) treatments were performed on ML-DmBG3-c2 cells using NEB (Ipswitch, MA, USA) protocols and buffers. Pelleted cells were resuspended in lysis buffer (Guy, 2000) [10 mM Hepes pH 7.6,1.5 mM MgCl2, 10 mM KCl, 250 mM sucrose, 5 mM EGTA, 5 mM EDTA, Protease Inhibitor (Calbiochem, San Diego, CA, USA)], flash frozen, thawed on ice, homogenized, and spun down (1000× ***g***, 5 min). Supernatant was transferred to new tubes, mixed with 10×Glycoprotein denaturing buffer, and put at 100°C for 10 min. This denatured solution was transferred to new tubes and 10×G5 was added to make a 1× and 1% NP40 final concentration. Endoglycosidase H was then added and tubes put at 37°C for 4 h, after which reactions were quenched with 6× sample buffer (βME+Protease Inhibitor) and prepared for western blots.

### Microscopy and data analysis

Specimens were viewed with constant acquisition settings on a Zeiss LSM700 Inverted confocal microscope. All methodology and statistics (including choice of sample size, exclusion criteria, double blind test, randomization, and choice of statistical test) were performed as previously described and according to standard procedures for this type of *Drosophila* data as described previously (Chu et al., 2006); e.g. quantification of growth cone, optic stalk and eye disc fluorescence. To count larval lamina neurons, stacks were normalized to the same threshold on a dark background using ImageJ. A smooth function eliminated scattered pixels. The area containing the lamina neuropil was highlighted and the ‘Analyze Particles’ function was used to give a count. ClustalW (EMBL-EBI) was used to align HhC from different species. Densitometry was performed using ImageJ. Protein molecular weight was estimated using the ‘Compute pI/Mw’ program (ExPASy).
